# Rotating Frame Relaxation Time Mapping for the Visualization of the Sinoatrial Node Without Contrast Agent

**DOI:** 10.1002/nbm.70083

**Published:** 2025-06-29

**Authors:** Yi Li, Victor Casula, Tarja Huhta, Sarah Mailhiot, Katja Tolkkinen, Jouni Karjalainen, Timo Liimatainen

**Affiliations:** ^1^ Research Unit of Health Sciences and Technology University of Oulu Oulu Finland; ^2^ Research Unit of Biomedicine and Internal Medicine University of Oulu Oulu Finland; ^3^ NMR Research Unit University of Oulu Oulu Finland; ^4^ Department of Radiology Oulu University Hospital Oulu Finland

**Keywords:** cardiac conduction system, fibrosis, magnetization transfer, myocardium, rotating frame relaxation, sinoatrial node

## Abstract

Late gadolinium‐enhanced cardiovascular magnetic resonance (LGE‐CMR) has been used to visualize the sinoatrial node (SAN) structure. In this study, we aimed to investigate the rotating frame relaxation (RAFF2 and T_1ρ_) time mapping to characterize the SAN structure in the heart ex vivo without a contrast agent. Ex vivo swine heart tissues were scanned on a 7 T vertical bore preclinical and a 3 T clinical MRI system. The contrast between the SAN and the surrounding atria was assessed using T_RAFF2_, T_1ρ_, T_1_, and T_2_ relaxation time mappings, as well as magnetization transfer (MT) imaging. Masson's trichrome stained histological sections were prepared to validate the location of the SAN. Relative relaxation time difference (RRTD) and MT contrast (MTC) were calculated for the SAN and remote myocardium areas. Significant differences between the SAN and myocardium were observed in all endogenous MRI contrasts. T_RAFF2_ and T_1ρ_ showed the highest contrast between the SAN and myocardium, although the differences in contrast among T_RAFF2_, T_1ρ_ and T_2_ were not significant at 3 T. Furthermore, SAN sizes determined based on T_RAFF2_ and T_1ρ_ maps were highly correlated with the corresponding fibrous structures in the histological sections. The 3D reconstruction of 2D T_RAFF2_ maps revealed the SAN region with increased relaxation time compared to the surrounding myocardium, located lateral to the crista terminalis. This study demonstrates the potential of T_RAFF2_ and T_1ρ_ for visualizing the SAN in the swine heart without the use of contrast agents. Detection of the SAN location using T_RAFF2_ and T_1ρ_ relaxation time mapping could offer a non‐invasive alternative to LGE‐CMR.

## Graphical abstract

In this study, we have found that SAN structure displays higher relaxation time compared to remote myocardium. T_RAFF2_ and T_1ρ_ are highly correlated with SAN size determined by histology. T_RAFF2_ and T_1ρ_ relaxation time maps are feasible non‐invasive methods to visualize SAN structure.

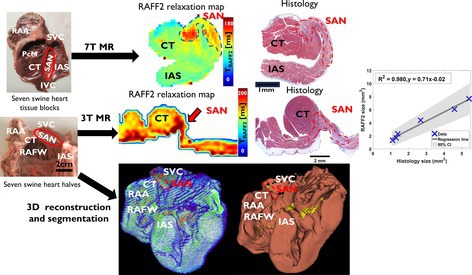

**Abbreviations**
ANOVA: Analysis of variance
AVA: Trial ventricle
CCS: Cardiac conduction system
CW: Continuous wave
CT: Crista terminalis
FOV: Field‐of‐view
Gd: Gadolinium
IAS: Interatrial septum
TI: Inversion time
LGE: Late gadolinium enhancement
LGE‐CMR: Late gadolinium enhancement cardiac MR
MT: Magnetization transfer
MTC: MT contrast
MTR: MT ratio
MESE: Multi‐echo spin echo
PBS: Phosphate‐buffered saline
RAFF: Relaxation along the fictious field
RAFFn: RAFF in the *n*th rotating frame
T_RAFF2_
: RAFF2 relaxation time
ROIs: Regions of interest
RRTD: Relative relaxation time difference
RAFW: Right atrial free wall
RA: Right atrium
SNR: Signal‐to‐noise ratio
SAR: Specific absorption rate
SL: Spin lock
TSL: Spin lock time
SVC: Superior vena cava

## 1 Introduction

The sinoatrial node (SAN), the uppermost part of the cardiac conduction system (CCS), is considered the main pacemaker of the heart, and it generates the electrical signal that causes the heart to contract [1, 2]. In 1907, Keith and Martin discovered the structure of the SAN [3], and it has since become an important research subject in cardiology and electrophysiology.

The structure and electrical activity of the SAN have been studied in clinical research as well as in animal models [4, 5]. The CCS of a swine heart closely resembles the human CCS [5]; however, it exhibits more connective tissue and less elastic tissue content [6]. The volume percentage of fibrotic connective tissue in the SAN of healthy adults is around 40%, while the amount of collagen and fibroblasts in the pig SAN is approximately 74% [6, 7]. Despite these differences, swine hearts can be used, with caution, as an experimental model to investigate human heart diseases [5, 6].

The SAN is located 1 mm beneath the epicardium in the right atrium, along the junction between the superior vena cava (SVC) and right atrium (RA), lateral to the crista terminalis (CT) [8]. It has an ellipsoidal fibrotic appearance, with a thickness of 2–3 mm and a length of 10–20 mm [8, 9]. Locating the SAN is important to avoid damaging it during surgery and to assess its functionality. Late gadolinium enhancement (LGE) has been used to visualize ventricular scars and myocardial fibrosis [10, 11], and together with cardiovascular magnetic resonance (LGE‐CMR), it aids in visualizing SAN fibrotic structure [12]. However, gadolinium (Gd) has its drawbacks, including its unsuitability for some patients, the risk of Gd accumulation in the brain, and contamination of natural water resources with Gd from MRI examinations [13, 14].

The connective tissue surrounding the SAN offers potential for visualizing the SAN through the contrast between the myocardium and fibrotic tissue. Longitudinal rotating frame relaxation time, T_1ρ_, has been introduced as a contrast agent‐free alternative to image myocardial infarction scar [15, 16, 17]. Compared to conventional T_1_ and T_2_, T_1ρ_ measured with on‐resonance RF irradiation is more selectively sensitive to certain slow molecular motions [18, 19]. However, the relatively high RF pulse powers and continuous wave irradiation of T_1ρ_ measurement can lead to a high specific absorption rate (SAR), which causes tissue heating. Relaxation along the fictious field (RAFF) and RAFF in the *n*th rotating frame (RAFFn) techniques have been developed to reduce the SAR by up to 90% compared to conventional T_1ρ_ measurement [20, 21]. In RAFF2, the fictitious field is produced by nested sine amplitude and cosine frequency‐modulated RF pulses [20, 21]. RAFF2 relaxation time (T_RAFF2_) is used to characterize the relaxation during the RF pulse, and has been applied to measure myocardial fibrosis in mice [17]. T_RAFF2_ has been shown to increase in myocardial fibrosis in a mouse model and in patients with myocardial infarction [19, 22].

Magnetization transfer (MT) imaging is more specific in detecting the amount of macromolecules in tissues than traditional MRI measures, including T_1_, T_2_, or proton density [23, 24, 25]. MT has been used in a wide range of applications in collagen and fibrosis detection, such as the evaluation of fibrosis in liver and intestinal tissue and the assessment of myocardial infarction [26, 27, 28, 29].

Here, we demonstrate contrast between the SAN and the surrounding myocardium without using a contrast agent. In this study, we aimed not only to leverage the advantages of a high‐field experimental scanner (offering high signal and resolution) but also to test the feasibility of imaging the SAN area using a 3 T clinical MRI scanner. For this purpose, we utilized T_1ρ_ and RAFF2 relaxation time maps and MT images to image ex vivo swine hearts at both 7 T and 3 T. A comparative analysis was conducted against conventional T_1_ and T_2_ maps and MT imaging. Masson's trichrome stained histology was used as a reference for identifying the fibrotic structure of the SAN.

## 2 Materials and Methods

### 2.1 Ex Vivo Sample Preparation for MRI

**FIGURE 1:**
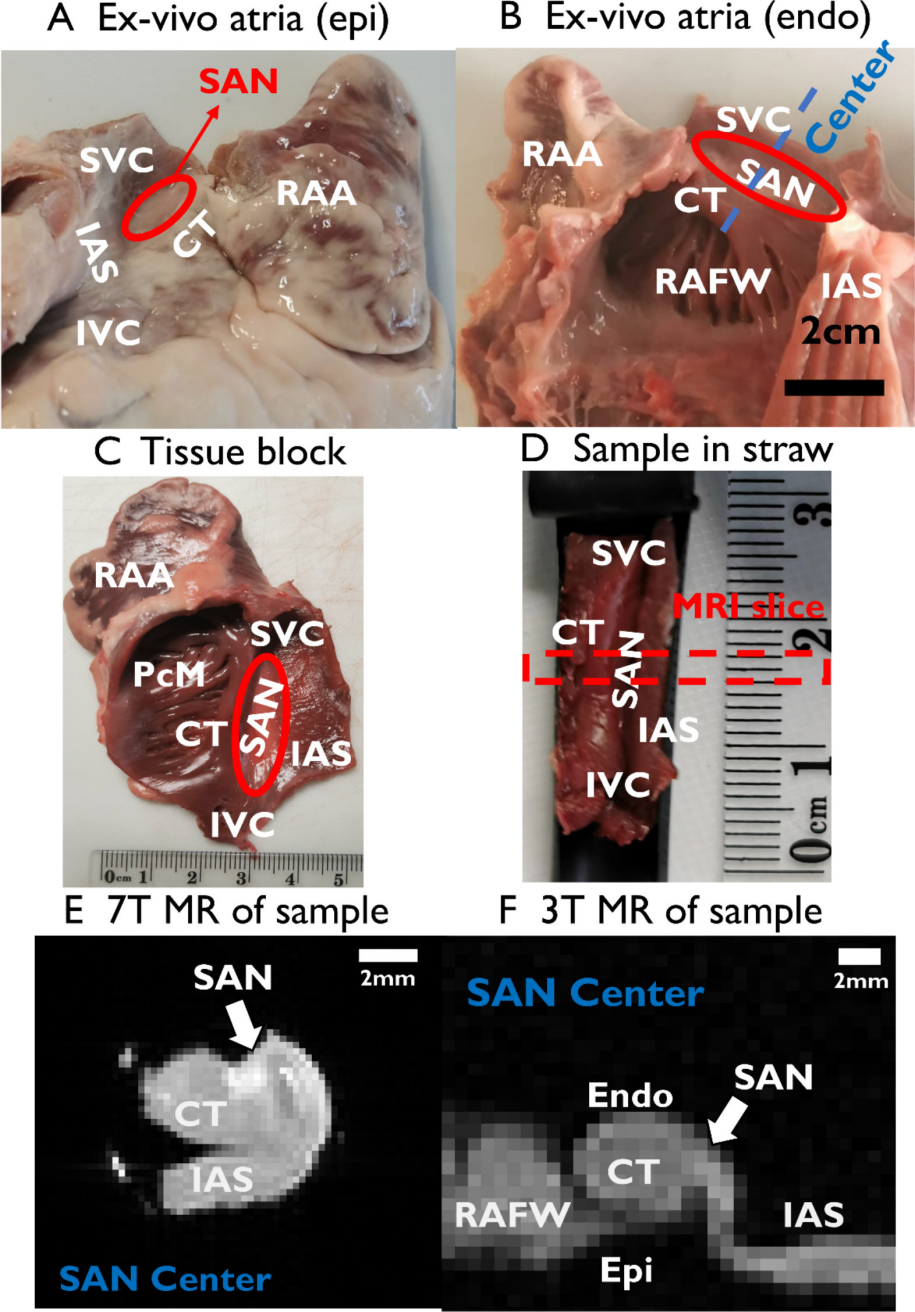
Preparation of the swine heart samples. (A) Photograph of the external structure of the right atrium. The approximate border of the SAN region is shown by a red oval. (B) Photograph of the internal structure of the right atrium. The approximate SAN region is shown by a red oval. The blue dashed line shows the SAN center region and the approximate position of the scanning plane. (C) Right atrium tissue block. The red oval shows the approximate location of the SAN. (D) The tissue is folded and stuck inside a plastic straw for imaging. (E) 7 T MR image of the SAN sample. The white arrow points to the center of SAN. (F) 3 T MR image of the SAN sample. The white arrow points to the center of the SAN. CT, crista terminals; Endo, endocardium; Epi, epicardium; IAS, interatrial septum; IVC, inferior vena cava; PcM, pectinate muscles; RAA, right atrial appendages; SAN, sinoatrial node; SVC, superior vena cava.

Ex vivo samples (*N* = 14) of swine hearts (Finnish Landrace, weighing 100 ± 5 kg), purchased from a local slaughterhouse, were stored at approximately 3–4 °C prior to MRI experiments. Seven hearts were longitudinally sectioned (Figure 1A,B) and scanned at 3 T. The remaining seven hearts were used to prepare tissue blocks (approximately 5 × 7 × 1 cm^3^) containing the SAN (Figure 1C), which were scanned at 7 T. Before the measurements, the samples were rinsed with phosphate‐buffered saline (PBS), marked at the SAN center, photographed, and transferred to the glass tube or plastic container. The samples were immersed in perfluoropolyether oil (Fomblin; Solvay Solexis, Italy) to provide a clean, ^1^H signal‐free background with magnetic susceptibility similar to that of tissue.

### 2.2 7 T MRI

The imaging of the tissue blocks was carried out at 7 T (Bruker III Avance 300, Bruker Biospin GmbH, Germany, vertical bore diameter 89 mm, maximum gradient strength 0.99 T/m) using a 10 mm quadrature RF volume transceiver (TX/RX). Each sample was placed in a plastic straw and then inserted into an NMR glass tube (Figure 1D) and positioned in the scanner with the SAN area aligned near the magnetic isocenter. A single 3 mm imaging slice was selected to cover the largest profile of the SAN structure (Figure 1E). RAFF2, T_1ρ_, T_2_, T_1_, and MT‐weighted images were acquired at room temperature with a resolution of 234 × 234 μm^2^, using an acquisition matrix of 64 × 64 and field of view (FOV) of 15 × 15 mm^2^, with a bandwidth of 50 Hz/pixel.

The RAFF2 pulse length was set according to the nominal maximum power of 1250 Hz to 2.26 ms [20], and the number of pulses was 0, 8, 16, and 32 (Supplemental Figure S1A). The T_1ρ_ measurements were performed with a rotating frame module (Supplemental Figure S1B), which contained a continuous wave (CW) spin lock pulse (power 1250 Hz) with four spin lock times (TSL) between 0 and 60 ms. A 90° excitation pulse was followed by two CW pulses having opposite polarities. Bipolar CW pulses were used to compensate for the spin lock field variations [30]. A 180° refocusing pulse between the CW halves was introduced to produce a spin echo, which compensates for B_0_ inhomogeneities [30, 31].

A multi‐echo spin‐echo (MESE) sequence was used to determine T_2_ relaxation time with four TEs: 11, 22, 33, 44 ms and a TR of 4 s. The fast spin echo (FSE) was used to determine T_1_ relaxation time with an inversion recovery mode, and the inversion time (TI) was set from 100 ms to 1200 ms to obtain five T1 weighted images. MT was measured using one CW pulse with a power of 425 Hz. Two offset frequencies (Δf) were used: 1500 Hz as the selected offset to assess the MT effect, and 20,000 Hz as the reference offset to represent the signal without significant MT saturation. The total scan time for one tissue block was approximately 2–3 h.

### 2.3 3 T MRI

The seven swine heart halves were scanned on a 3‐T MRI scanner (MAGNETOM Vida, Siemens Healthineers AG, Erlangen, Germany) using an 18‐channel Tx/Rx knee coil. Each specimen was fixed in a plastic container filled with perfluoropolyether and then placed in the center of the coil. Multiple 2 mm thick slices perpendicular to the CT were selected, covering most parts of the swine heart sample, including the SAN, surrounding CT, interatrial septum (IAS), and right atrial free wall (RAFW). The slice including the largest profile of the SAN structure was selected based on the 3D T1‐weighted images (Supplemental Methods). RAFF2, T_1ρ_, T_2_, T_1_, and MT were acquired with a resolution of 0.6 × 0.6 mm^2^, an acquisition matrix of 192 × 156, FOV of 111 × 90 mm^2^, and a bandwidth of 260 Hz/pixel.

The maximum RF pulse power for RAFF2 was set to 500 Hz, and the duration of the composite pulse was set to 2.82 ms (pulse trains of 0, 8, 16, 24, and 32 pulses). The T_1ρ_ measurements were performed with a CW pulse power of 500 Hz and five TSL between 0 and 60 ms. For T_2_, TE was set from 10 to 50 ms in steps of 10 ms. T_1_ was obtained with the application of a saturation pulse (six TI between 200 and 3900 ms). MT was measured using a CW pulse with a power of 500 Hz. Two offset frequencies (Δf) were applied: 1000 Hz to assess the MT effect, and 50,000 Hz as the reference offset to represent the signal without significant MT saturation. The total acquisition time for all the sequences is around 8 to 10 h. The other sequence parameters can be found in the Supplemental Methods.

### 2.4 Histological Validation of SAN

After MRI, 5‐μm‐thick histology sections were prepared from the sample block. The sample was cut longitudinally, and a cross section near the center was selected. The cross sections were stained with Masson's trichrome, where collagen‐rich tissue appears blue and myocytes are shown in red. Photographs were acquired using an optical microscope equipped with a digital camera (Nikon Eclipse Ni‐E, Tokyo, Japan). Further details regarding the histological procedures are provided in the Supplemental Methods.

### 2.5 Data Analysis

Relaxation time maps were calculated and reconstructed from signal intensity images with pixel‐by‐pixel analysis using the Aedes software package (http://aedes.uef.fi/) in MATLAB (Mathworks Inc., Natick, Massachusetts, United States). The RAFF2 relaxation time map was created from the weighted images by fitting a two‐parameter signal model, $S\left(t\right)=S_{0}e^{\left(-\frac{t}{T_{\text{RAFF}2}}\right)}$, where *S*_0_ is the initial magnetization. Other relaxation time maps were fitted using an equivalent mono‐exponential model.

To assess the agreement between relaxation time maps and histology images, 2D histological images were analyzed in MATLAB at a resolution of 200 × 200 μm^2^, using the blue channel of the raw RGB images to distinguish fibrotic content. We have applied the Otsu's threshold method, which is an automatic thresholding algorithm, and compared the histological images with the corresponding 2D cross sections of relaxation time maps from the same location [32]. The SAN region includes the surrounding myocardial tissue of CT, IAS, RAFW, and the epicardial fibrotic layer [33]. Based on the composition of the SAN sample, the number of thresholds (*N* = 3) of multi‐Otsu thresholding method was selected to divide the histological image into four regions, which are the background, myocardium, SAN and epicardial fibrotic layer. The relaxation time maps were analyzed and divided into four regions based on the same principle as the histology image (Supplemental Figures S2 and S3). The SAN relaxation time threshold was then applied to the segmentation of the SAN region in the 3D relaxation time map. The segmented histology image and relaxation time maps were further used to define the regions of interest (ROIs) by cropping the desired portion of the image. SAN sizes in the histology images and corresponding relaxation time maps were calculated as the area (number of pixels) of the SAN in the segmented results. The correlation between the SAN sizes derived from the relaxation time maps and histology was evaluated using Pearson correlation analysis. *p* Values < 0.05 were considered statistically significant. Bland–Altman analysis was also used to assess the agreement between the SAN sizes determined by histology and relaxation time maps. The mean and difference of each pair were calculated, and the bias and 95% limits of agreement (mean ± 1.96 × SD) were determined.

Relaxation times were averaged on the defined ROIs. The contrast in the relaxation time maps was defined as the relative relaxation time difference (RRTD), calculated as follows: RRTD = 2[T (SAN)‐T (myocardium)]/[T (SAN) + T (myocardium)], where T (SAN) and T (myocardium) are the average relaxation times (T_RAFF2_, T_1ρ_, T_2_, or T_1_) in the SAN and the remote myocardium area, respectively. The MT ratio (MTR) was calculated as MTR = 100 × (S_0_‐S_MT_)/S_0_, where *S*_0_ is the signal within the defined ROIs with 20,000 Hz offset, and *S*_MT_ is the signal within the same ROIs with the selected offset. The MT contrast (MTC) between the SAN and myocardium was calculated using MTC = 2[MTR (myocardium)‐MTR (SAN)]/[MTR (SAN) + MTR (myocardium)].

The 2D RAFF2 relaxation time maps derived from 3 T MR measurements were smoothed, volume‐rendered, and reconstructed into a 3D visualization using the ImageJ plugin, Volume Viewer (http://rsb.info.nih.gov/ij/plugins/volume‐viewer.html) [34]. The SAN threshold was applied to segment different regions of SAN, CT, IAS, and RAFW. The segmentation of the SAN region was performed and visualized in 3D using both MATLAB and 3D Slicer (http://www.slicer.org) [35].

B_1_ maps were obtained by fitting the cosine function to the data acquired by varying the length of a hard pulse from 0 to 0.36 ms, using the same power (1250 Hz at 7 T and 500 Hz at 3 T) applied in other relaxation time measurements. B_0_ maps were obtained based on the phase change between gradient echo with different echo times. B_1_ homogeneity was verified to be within ± 10% Hz, and the variation in B_0_ was less than ± 100 Hz across the entire myocardium.

All the continuous variables are presented as mean ± standard deviation. All statistical analyses were performed using the MATLAB Statistics Toolbox. A normality test (Shapiro–Wilk) was conducted to confirm the normal distribution of the data. An independent t‐test was applied to compare the mean difference between the SAN and myocardium areas of each imaging technique. A one‐way ANOVA with Benjamini–Hochberg correction for multiple comparisons was used to determine the significance of the differences in contrast values between the RAFF2 and other imaging methods.

## 3 Results

**FIGURE 2:**
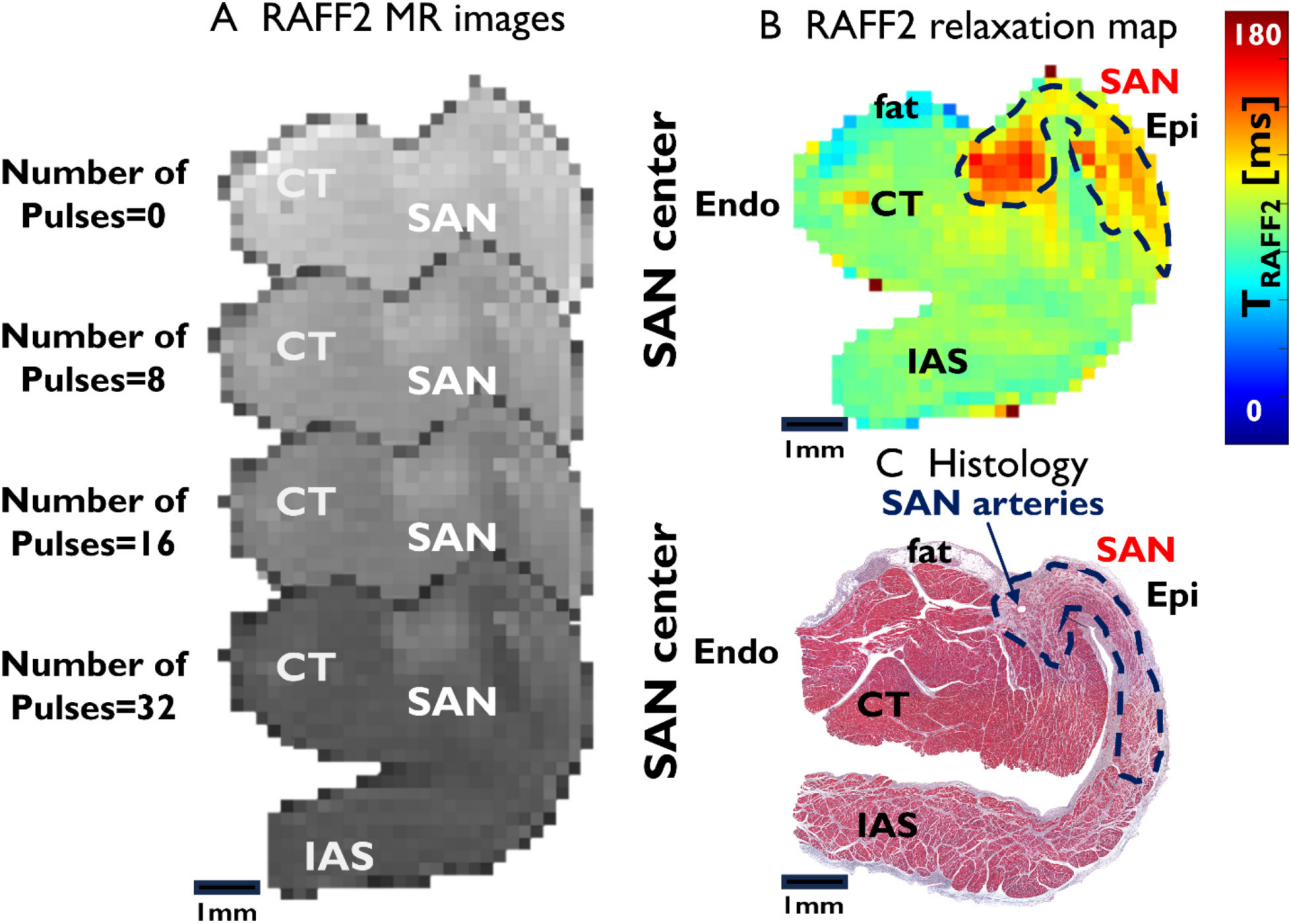
(A) Examples of RAFF2 images obtained for a swine heart sample at 7 T with 0, 8, 16, and 32 weighting pulses. (B) RAFF2 relaxation time map from the SAN center, T_RAFF2_. The map shows the SAN as a clear structure distinct from the surrounding myocardium. The approximate border of the SAN center is shown by the blue dashed line. (C) Corresponding histology section (Masson's trichrome). The blue arrow points to the approximate region of the SAN artery. The ROIs are chosen according to the information in the histology image. CT, crista terminalis; Endo, endocardium; Epi, epicardium; IAS, interatrial septum; SAN, sinoatrial node.

**FIGURE 3:**
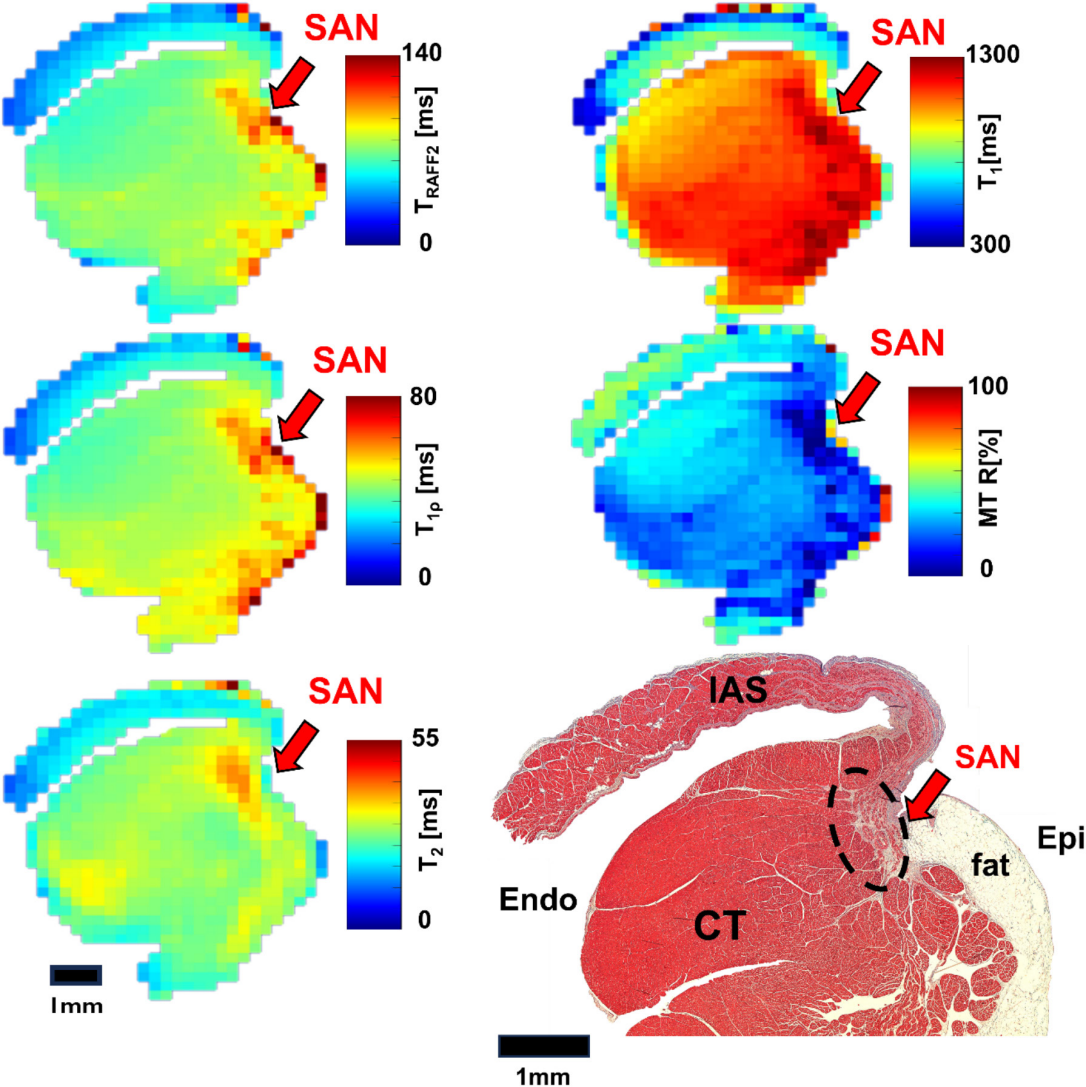
Example of T_RAFF2_, T_1ρ_, T_2_, and T_1_ relaxation time maps, and MTR obtained in a swine heart sample at 7 T, and the corresponding histology image. The red arrows point to the SAN region.

In the RAFF2‐weighted images acquired at 7 T MRI, the SAN region demonstrates increased signal intensity adjacent to the CT, providing clear contrast with the surrounding myocardium (Figure 2A). As T_RAFF2_ weighting increases, the signal loss in the myocardium becomes more evident than in the SAN. The RAFF2 maps show that SAN exhibits longer relaxation times (123.1 ± 13.3 ms) compared to the myocardium (91.6 ± 8.5 ms) (Figure 2B). The corresponding histological image confirms the approximate SAN location and reveals the presence of the SAN artery within the region (Figure 2C). ROIs for both SAN and myocardium were defined using segmented histological data and relaxation time maps. The contrast between SAN and myocardium can be observed in the T_RAFF2_, T_1ρ_, and T_2_ maps, as well as in MTR images (Figure 3). Statistically significant differences are found between the SAN and myocardium across all imaging methods (**p < 0.01, ***p < 0.001; Table 1). RAFF2 and T_1ρ_ exhibit significantly greater contrast between the SAN and myocardium compared to T_1_, T_2_, and MT (^#^ *p* < 0.05, ^###^ *p* < 0.001; Table 1, details shown in Supplemental Figure S4).

**TABLE 1:** Relaxation times and MTR in SAN and in myocardium (mean ± standard deviation) from 7 T with corresponding contrast (RRTD and MTC).

MRI contrast	Regions of interest		Contrast (RRTD/MTC)
	SAN	Myocardium	
T_RAFF2_ (ms)	124 ± 15	90 ± 8***	0.31 ± 0.06
T_1ρ_ (ms)	60 ± 10	43 ± 5**	0.33 ± 0.15
T_2_ (ms)	47 ± 7	36 ± 7***	0.27 ± 0.10^#^
T_1_ (ms)	1322 ± 106	1166 ± 79**	0.12 ± 0.02^###^
MTR (%)	36 ± 5	47 ± 6***	0.26 ± 0.03^#^

Statistical significance (independent *t* test after Benjamini–Hochberg correction) is indicated as **p < 0.01, ***p < 0.001 for differences between SAN and myocardium, and ^#^ *p* < 0.05, ^###^ *p* < 0.001 for differences in the contrast using RAFF2 as the reference.

**FIGURE 4:**
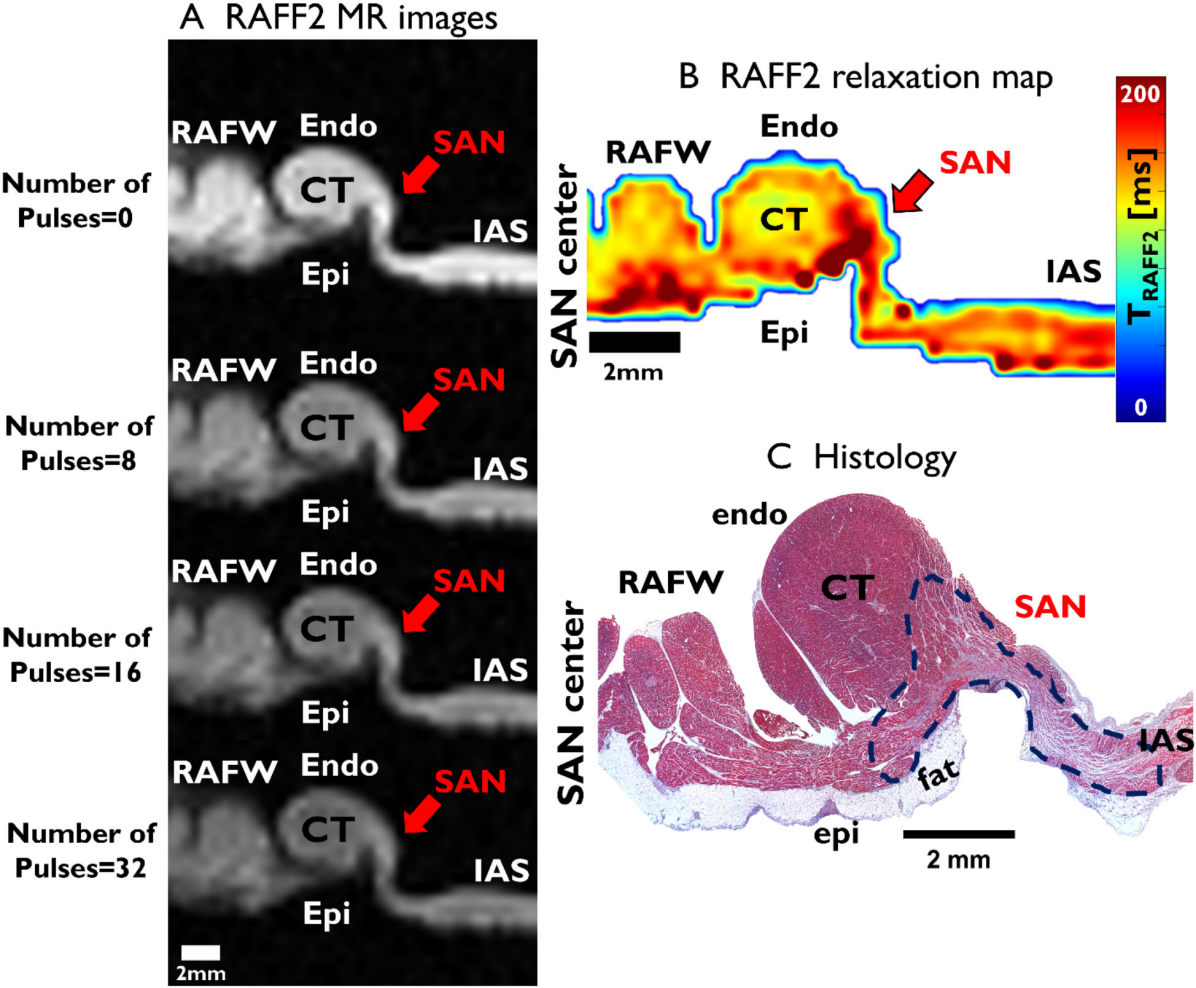
(A) RAFF2‐weighted image series (3 T MRI) obtained with 0, 8, 16, and 32 RAFF2 pulses. The red arrow points to the SAN center. (B) RAFF2 relaxation time map from the SAN center, T_RAFF2_. The map shows the SAN as a clear structure distinct from the surrounding myocardium. The red arrow points to the SAN region. (C) Corresponding histology section (Masson's trichrome). The approximate border of the SAN center is shown by the blue dashed line. CT, crista terminals; Endo, endocardium; Epi, epicardium; IAS, interatrial septum; RAFW, right atrial free wall; SAN, sinoatrial node.

**FIGURE 5:**
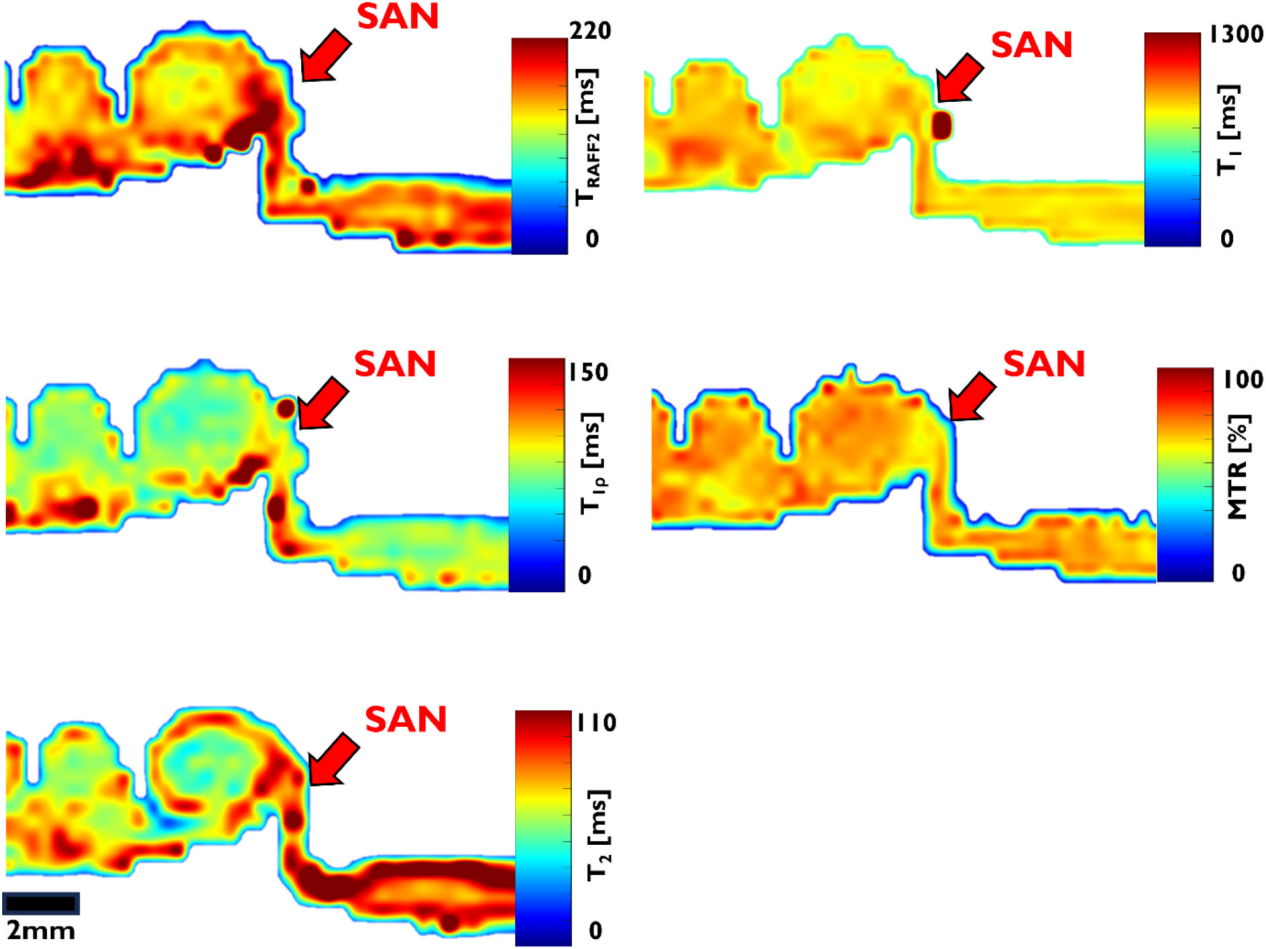
Example of T_RAFF2_, T_1ρ_, T_2_, and T_1_ relaxation time maps, and MTR of SAN and surrounding myocardium in a swine heart obtained at 3 T. The red arrows point to the SAN region.

Increasing T_RAFF2_ weighting enhances contrast between the SAN and myocardium in 3 T MRI (Figure 4A). The RAFF2 maps show an area of elevated relaxation time (190 ± 10 ms) than the remote myocardium (90 ± 8 ms), corresponding to the approximate structure of the SAN delineated in histological images (Figure 4B,C). The T_RAFF2_, T_1ρ_, and T_2_ maps demonstrate more pronounced contrast between the SAN area and surrounding myocardium than the T_1_ map and MTR (Figure 5). The values of all relaxation times and MTR are significantly elevated in the SAN area compared to the myocardium (***p < 0.001, **p < 0.01; Table 2, details shown in Supplemental Figure S5). The contrast (RRTD) in T_RAFF2_ is significantly greater than the contrast (MTC) in MTR (^##^ *p* < 0.01) and the RRTD in T_1_ (^###^ *p* < 0.001). No significant differences are found in RRTD among T_RAFF2_, T_1ρ_, and T_2_.

**TABLE 2:** Relaxation times and MTR in SAN and in myocardium (mean ± standard deviation) from 3 T MRI and corresponding contrast (RRTD and MTC).

MRI contrast	Regions of interest		Contrast (RRTD/MTC)
	SAN	Myocardium	
T_RAFF2_ (ms)	193 ± 13	130 ± 3***	0.40 ± 0.06
T_1ρ_ (ms)	113 ± 6	75 ± 3***	0.39 ± 0.05
T_2_ (ms)	97 ± 16	60 ± 8***	0.46 ± 0.11
T_1_ (ms)	1074 ± 117	945 ± 78**	0.13 ± 0.06^###^
MTR (%)	56 ± 6	67 ± 7***	0.17 ± 0.02^###^

Statistical significance (independent *t* test after Benjamini–Hochberg correction) is indicated as **p < 0.01, ***p < 0.001 for differences between SAN and myocardium, and ^###^ *p* < 0.001 for differences in the contrast using RAFF2 as the reference.

**FIGURE 6:**
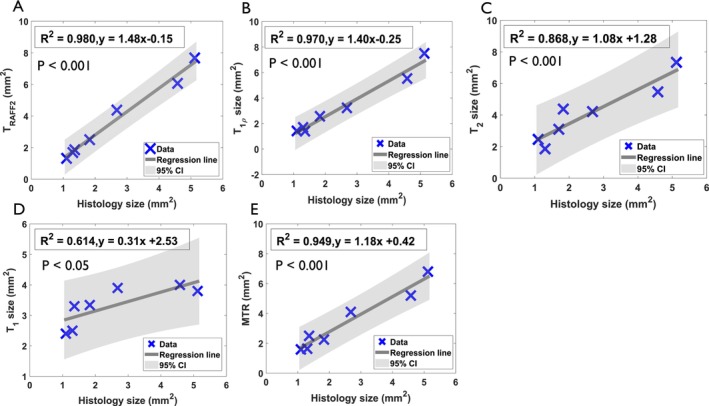
Linear correlation between the SAN areas derived from 7 T MRI T_RAFF2_ (A), T_1ρ_ (B), T_2_ (C), T_1_ (D), MTR (E) and Masson's trichrome stained histology sections. Pearson's correlation coefficient of determination with significance level P and formulas of linear relationship are shown next to the fitted correlation line.

The agreement between several relaxation time maps at 7 T and histology is observed in the measurement of SAN areas (Figure 6). At 7 T MRI, the strongest Pearson correlations are obtained with T_RAFF2_ (*R* ^2^ = 0.98, *p* < 0.001) and T_1ρ_ (*R* ^2^ = 0.97, *p* < 0.001). SAN areas derived from MTR (*R* ^2^ = 0.949, *p* < 0.001) and T_2_ (*R* ^2^ = 0.868, *p* < 0.01) also show strong correlations with histology, while T_1_‐derived SAN areas exhibit a comparatively moderate correlation (*R* ^2^ = 0.614, *p* < 0.05) (Figure 6). At 3 T, SAN sizes derived from T_RAFF2_ (*R* ^2^ = 0.858, *p* < 0.05), T_1ρ_ (*R* ^2^ = 0.849, *p* < 0.05), and T_2_ (*R* ^2^ = 0.804, *p* < 0.05) show statistically significant correlations with SAN sizes measured from histology sections (Supplemental Figure S6). The SAN sizes derived from T_1_ and MTR do not correlate with histology. Bland–Altman plots demonstrate that SAN sizes measured by all relaxation time maps systematically overestimate those measured by histology (Supplemental Figure S7). At 7 T, T_2_ shows the greater overestimation, with a mean bias of +1.55 mm^2^ and wide limits of agreement (LOA +0.18 to +2.92 mm^2^). In contrast, T_RAFF2_, T_1ρ_, and MT show smaller overestimation, with mean biases of +1.07 mm^2^ (LOA −0.62 to +2.76 mm^2^), +0.77 mm^2^ (LOA −0.52 to +2.06 mm^2^), and +0.87 mm^2^ (LOA −0.4 to +2.14 mm^2^), respectively. The lower biases and narrower LOAs indicate better agreement with histology. At 3 T, Bland–Altman analysis shows good agreement between histology and SAN sizes from T_RAFF2_ (mean bias +0.64 mm^2^, LOA −0.25 to +1.52 mm^2^) and T_1ρ_ (mean bias −0.67 mm^2^, LOA −1.90 to +0.55 mm^2^) (Supplemental Figure S8). In comparison, T_2_ (mean bias +1.83 mm^2^, LOA −0.35 to +4.02 mm^2^), T_1_ (mean bias −0.88 mm^2^, LOA −3.01 to +1.25 mm^2^), and especially MT (mean bias +4.79 mm^2^, LOA +1.26 to +8.31 mm^2^) show larger biases and wider LOAs, indicating greater discrepancies from histology. These results suggest that T_RAFF2_ and T_1ρ_ provide more accurate estimates of SAN size at both 7 T and 3 T.

**FIGURE 7:**
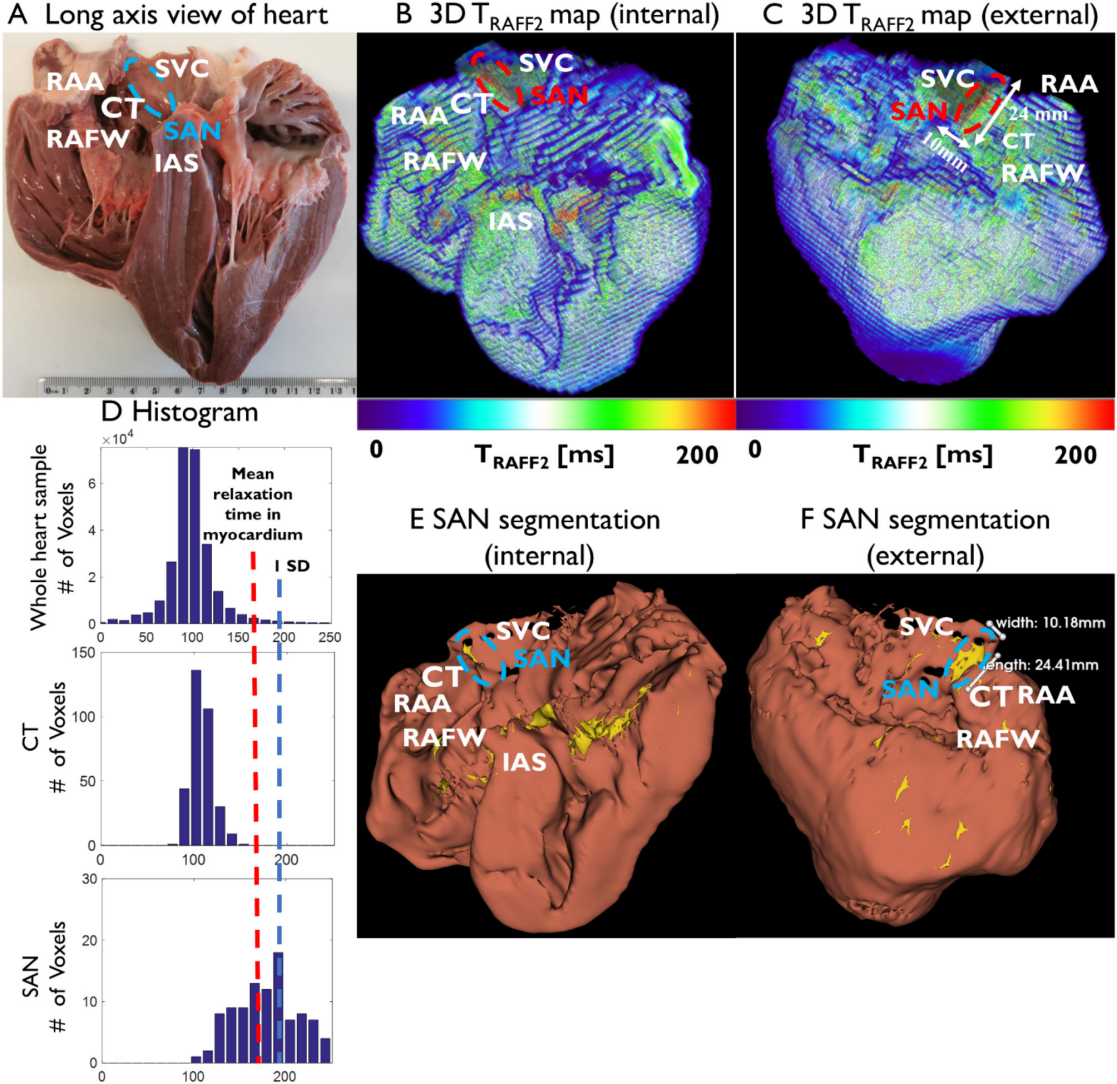
3D SAN structure identification by the RAFF2 relaxation time mapping. (A) Photograph of the long axis view of ex vivo heart used for 3 T MRI. The blue dashed outline shows the SAN region. (B) Internal and (C) external views of the 3D T_RAFF2_ map reconstructed from 2D T_RAFF2_ maps. The red dashed outline and white arrows show the approximate position and size of the SAN region. (D) Relaxation time histograms in different swine heart structures. The threshold (20% higher than the mean myocardium relaxation time) to segment the myocardium and the SAN is indicated with the red line. (E,F) Segmentation of the SAN and other fibrotic structures, as well as the myocardium, is illustrated in the internal and external views of the 3D reconstructed structure. CT, crista terminals; IAS, interatrial septum; RAA, right atrial appendages; RAFW, right atrial free wall; SVC, superior vena cava; SAN, sinoatrial node.

Based on the multi‐Otsu thresholding results, the optimal SAN thresholds for T_RAFF2_ and T_1ρ_ relaxation times are approximately 20% and 15% higher than the average myocardium relaxation time, respectively. The structure of the SAN can be delineated in the 3D reconstructed RAFF2 relaxation time maps of the ex vivo hearts by distinguishing a significantly higher relaxation times in the SAN compared to the surrounding myocardial tissue (Figure 7A–C). After removing the background from the relaxation time maps, histograms of relaxation times for the whole heart sample, CT, and SAN were generated and compared (Figure 7D). The difference in relaxation time allows discrimination of the SAN and other highly fibrotic regions from the myocardium in the 3D‐reconstructed structure (Figure 7E,F).

## 4 Discussion

The SAN structure was successfully visualized without a contrast agent in ex vivo swine hearts using rotating frame relaxation time maps. The identification of the SAN at both 7 T and 3 T MRI was validated against histological sections of the SAN of the same ex vivo swine heart. SAN sizes measured from T_RAFF2_ and T_1ρ_ relaxation time maps showed a high correlation with those determined from histology sections. Eliminating the drawbacks of contrast agents in visualizing the SAN structure has the potential to enhance the diagnosis of SAN dysfunction and other SAN‐related diseases.

T_RAFF2_ and T_1ρ_ exhibited high contrast between the SAN and surrounding myocardium in both 7 T micro‐MRI and 3 T clinical MRI. Bland–Altman plots show that areas of elevated T_RAFF2_ and T_1ρ_ closely correspond to SAN areas identified in histology. These results, along with the strong correlation between T_RAFF2_, T_1ρ_, and histology‐derived SAN sizes, demonstrate that rotating frame relaxation time maps (T_RAFF2_ and T_1ρ_) can detect SAN areas with high accuracy.

As expected, T_1_ relaxation times were shorter at 3 T [36], while T_RAFF2_, T_1ρ_ and T_2_, in both the myocardium and the SAN, were longer at 3 T compared to 7 T. Although higher field strength provides an improved signal‐to‐noise ratio (SNR), susceptibility effects, B_1_ and B_0_ inhomogeneities, as well as microscopic diffusion, are also accentuated, shortening the relaxation time [37]. Chemical exchange and molecular diffusion may be efficient contributors to relaxation at 7 T and cause the reduction in T_2_, T_1ρ_, and T_RAFF2_ relaxation times compared to 3 T [38, 39].

Multiple relaxation time maps were compared in ex vivo swine heart samples at 7 T and 3 T. At 7 T, RAFF2 showed higher RRTD, and SAR was approximately 60% lower than in T_1ρ_ [20]. This makes RAFF2 less restricted by SAR in clinical patient measurements, potentially offering a higher contrast than T_1ρ_ between SAN and remote myocardium tissue with acceptable SAR values. The SAN of both human and swine hearts exhibits large amounts of fibrotic connective tissue, collagen, and fibroblasts [6, 7]; therefore, our results align with previous studies in mouse and human hearts that associate elevated T_RAFF2_ and T_1ρ_ with areas of high fibrosis content in myocardial infarcts compared to remote myocardium areas [16, 18, 22].

In addition to the large amount of fibrotic content, the volume percentage of extracellular space in healthy adult human SAN is approximately 36%, which is much higher than that in remote myocardium. Higher LGE signal intensity has been shown in the SAN than surrounding tissue due to the accumulation of gadolinium contrast in highly fibrotic tissue with increased extracellular space [18].The observed elevations in T_1ρ_ and T_RAFF2_ within the SAN may be attributed to expanded extracellular space and increased tissue water content, consistent with findings in fibrotic myocardial infarct tissue [17]. Changes in pH or proton chemical exchange between water and macromolecules may also change T_1ρ_ relaxation time [16, 18, 40].

T_2_ in the SAN was significantly higher than in the remote myocardium area. T_2_ has been shown to have a strong correlation with myocardial water content in previous studies in animals and patients with myocardial diseases [40, 41]. However, in our estimates of SAN size from 7 T MRI, the correlation with histology was lower for T_2_ compared to T_RAFF2_, T_1ρ_, and MTR. Additionally, T_2_ maps tended to overestimate SAN sizes compared to T_RAFF2_, T_1ρ_ and MTR.

MT has been used to investigate the fibrosis in swine kidneys, collagen fibers in plaque and fibrin, and Purkinje fibers in pig hearts [26, 27, 28, 29].MT can be used to facilitate the contrast between SAN and myocardium, the latter containing little MT effects [27, 29]. In addition, MT may offer specificity to a chemical group in myocardial tissue due to its off‐resonance RF pulse preparation [42]. We found that MTC between SAN and myocardium was lower than the RRTD in relaxation time maps. The SAN areas derived from MTR showed a significant correlation with SAN size from histology at 7 T MRI, but not at 3 T. Furthermore, the contrast in MTR was significantly lower than the RRTD in T_RAFF2_ and T_1ρ_ at 3 T. Overall, T_RAFF2_ and T_1ρ_ overperformed MTR in revealing SAN area at 3 T.

There are several limitations in this study. Firstly, all measurements were conducted on ex vivo swine hearts. Although the pig heart is mainly similar to the human heart in size and structure, there are some differences in the SAN structure [5]. Secondly, the sample size is relatively small: seven samples for 7 T and seven for 3 T. Thirdly, the proposed method requires further validation by comparing normal and pathological SAN, which could be investigated using animal models of SAN‐related diseases [43, 44, 45]. However, our findings suggest an association between fibrotic content in myocardial tissue and the relaxation times T_RAFF2_ and T_1ρ_.Therefore, the methods presented in this study may offer better contrast between myocardium and SAN in case of pathological increase in fibrosis, particularly in sinoatrial node dysfunction conditions such as atrial fibrillation and heart failure, where SAN pacemaker cells are replaced by fibrotic tissue [43, 44, 45, 46, 47]. Fourthly, ex vivo hearts are blood‐free, which may affect the contrast between SAN and myocardium.

The high spatial resolution used in this work poses an additional challenge for applying the presented methods to image the SAN in humans. Although some accuracy is lost, it can be demonstrated that the methods remain applicable at lower resolutions (Supplemental Figure S9).

Future work should explore the diagnostic potential of non‐contrast‐enhanced MRI of the SAN, expanding these findings to human SAN imaging in healthy subjects and patients with SAN dysfunction. In this study, we selected Masson's trichrome staining for its ability to clearly differentiate between tissue components; however, Sirius Red, another sensitive stain for collagen detection, could be used in future studies to investigate different collagen types [48, 49]. T_RAFF2_ and T_1ρ_ may also facilitate imaging of other CCS structures, such as the atrioventricular (AV) node, bundle of His, and Purkinje fibers, due to their high fibrosis content.

## 5 Conclusion

The SAN structure can be distinguished from the myocardium by the longer rotating frame relaxation time values. Of the five MRI parameters tested, T_RAFF2_ and T_1ρ_ showed the highest contrast at 7 T and were comparable to T_2_ at 3 T. SAN sizes determined from T_RAFF2_ and T_1ρ_ correlated significantly with histology. T_RAFF2_ and T_1ρ_ mappings are feasible non‐invasive methods to identify the SAN structure in the swine heart ex vivo. Visualization of the SAN structure by rotating frame relaxation time maps (T_RAFF2_ and T_1ρ_) holds potential as a non‐invasive alternative to LGE‐CMR for in vivo applications.

## Author Contributions

Y.L. participated in the design of this study, prepared the heart samples, acquired the images, performed all the data analysis, and wrote the original manuscript draft. V.C. participated in the experiments, reviewed the manuscript, and provided feedback to the manuscript. T.H. completed all the histology sections staining. T.L. conceptualized, designed, and coordinated the study, and reviewed the manuscript. All authors edited, reviewed and approved the final manuscript. Open access publishing facilitated by Oulun yliopisto, as part of the Wiley ‐ FinELib agreement.

## Conflicts of Interest

The authors declare no conflicts of interest.

## Ethics Approval and Consent to Participate

Not applicable.

## Supporting information


**Figure S1** (A) Simplified schematic of the RAFF2 sequence, consisting of the preparation module and a read‐out. (B) T1ρ sequence. The rotating frame preparation module consists of two 90° RF pulses with opposite phases and a 180° refocusing pulse between the spin lock halves.
Figure S2 Correlation between the SAN areas in the histology image (A) and the corresponding RAFF2 relaxation time map (B). The blue channel of the raw RGB histology image is shown in a grayscale image. Based on the histogram of the images and optimal thresholds obtained from multiOtsu thresholding, the pixels were divided into four classes, which are background, myocardium, SAN, and epicardial fibrotic layer. The size of SAN was quantified by the area of SAN.
Figure S3 Histology images, corresponding TRAFF2 relaxation time maps, and SAN area segmentation results of seven representative samples which were scanned at 7 T.
Figure S4 Relaxation times of the 7 T MRI data. Contrast between SAN and myocardium was calculated by RRTD. Statistical significance (independent *t* test after Benjamini–Hochberg correction) is indicated as **P.
Figure S5 Relaxation times of the 3 T MRI data. The contrast between SAN and myocardium was calculated using RRTD. Statistical significance (independent *t* test after Benjamini–Hochberg correction) is indicated as **P.
Figure S6 Linear correlation between the SAN areas determined by 3 T MRI TRAFF2 (A), T1ρ (B), T2 (C), T1 (D), MTR (E), and Masson's trichrome stained histology sections. R^2^ indicates the Pearson correlation coefficient of determination with a significance level P.
Figure S7 Bland–Altman plots showing the agreement between SAN sizes determined by histology and 7 T MRI TRAFF2 (A), T1ρ (B), T2 (C), T1 (D), MTR (E). Each plot displays the mean difference (bias) as a solid red line and the 95% limits of agreement (mean ± 1.96 × SD) as dashed black lines.
Figure S8 Bland–Altman plots showing the agreement between SAN sizes determined by histology and 3 T MRI TRAFF2 (A), T1ρ (B), T2 (C), T1 (D), MTR (E). Each plot shows the mean difference (bias) as a solid red line and the 95% limits of agreement (mean ± 1.96 × SD) as dashed black lines.
Figure S9 (A) TRAFF2 relaxation time map derived from high resolution (0.6 mm) RAFF2 weighted images, segmentation and quantification results of one representative sample at 3 T, and (B) relaxation time map obtained from down sampling weighted images, segmentation and quantification results of the same sample.

## Data Availability

The data underlying this article will be shared on reasonable request to the corresponding author.
