# Supplementary material for: Rotating Frame Relaxation Time Mapping for the Visualization of the Sinoatrial Node Without Contrast Agent
Source: NMR Biomed. 2025 Jun 29;38(8):e70083. doi: 10.1002/nbm.70083 (PMC12206955; doi:10.1002/nbm.70083)
Supplement: Supplementary file 1 — Figure S1 (A) Simplified schematic of the RAFF2 sequence, consisting of the preparation module and a read‐out. (B) T1ρ sequence. The rotating frame preparation module consists of two 90° RF pulses with opposite phases and a 180° refocusing pulse between the spin lock halves. Figure S2 Correlation between the SAN areas in the histology image (A) and the corresponding RAFF2 relaxation time map (B). The blue channel of the raw RGB histology image is shown in a grayscale image. Based on the histogram of the images and optimal thresholds obtained from multiOtsu thresholding, the pixels were divided into four classes, which are background, myocardium, SAN, and epicardial fibrotic layer. The size of SAN was quantified by the area of SAN. Figure S3 Histology images, corresponding TRAFF2 relaxation time maps, and SAN area segmentation results of seven representative samples which were scanned at 7 T. Figure S4 Relaxation times of the 7 T MRI data. Contrast between SAN and myocardium was calculated by RRTD. Statistical significance (independent t test after Benjamini–Hochberg correction) is indicated as **P. Figure S5 Relaxation times of the 3 T MRI data. The contrast between SAN and myocardium was calculated using RRTD. Statistical significance (independent t test after Benjamini–Hochberg correction) is indicated as **P. Figure S6 Linear correlation between the SAN areas determined by 3 T MRI TRAFF2 (A), T1ρ (B), T2 (C), T1 (D), MTR (E), and Masson's trichrome stained histology sections. R2 indicates the Pearson correlation coefficient of determination with a significance level P. Figure S7 Bland–Altman plots showing the agreement between SAN sizes determined by histology and 7 T MRI TRAFF2 (A), T1ρ (B), T2 (C), T1 (D), MTR (E). Each plot displays the mean difference (bias) as a solid red line and the 95% limits of agreement (mean ± 1.96 × SD) as dashed black lines. Figure S8 Bland–Altman plots showing the agreement between SAN sizes determined by histology and 3 T MRI TRAFF [file NBM-38-e70083-s001.pdf]

Supplementary information for “Rotating frame relaxation time mapping for the visualization of the sinoatrial node without contrast agent”

Yi Li, Victor Casula, Tarja Huhta, Sarah Mailhiot, Katja Tolkkinen, Jouni Karjalainen, Timo Liimatainen

Supplemental Methods

7T MRI

$T_{1\rho}$ and T_{RAFF2} measurements were performed using a gradient echo readout sequence with a repetition time (TR) of 4000 ms, echo time (TE) of 5.5 ms, and a flip angle of 25 °. T_1, T_2 and MT measurements were performed with TR = 5000 ms and TE = 5.5 ms. MT offset frequency was 1500 Hz.

3T MRI

The parameters of the 3D T1-weighted images were TE = 2 ms, TR = 241.74 ms, resolution = 0.53 × 0.53 mm^2, matrix size = 220 × 320, FOV = 116 × 169 mm^2 slice thickness = 0.5 mm. The RAFF2 measurements were performed using a gradient echo readout with TR = 3 s, TE= 4.86 ms, number of averages = 5, and a flip angle of 25 °. T1 mapping was performed with TR = 4.09 s and TE = 9.6 ms, using an inversion recovery. T2-weighted images were acquired with TR = 3 s. For MT imaging, the specific sequence parameters were TR = 5 s, TE = 5.45 ms, number of averages = 1, and offset frequency = 1000 Hz.

Histological validation of SAN

After the measurements, all *ex vivo* swine heart sample blocks were perfused with phosphate buffered saline (PBS) and then immersion fixed with 4% formaldehyde. The samples were dehydrated with alcohol, cleared with xylene, and infiltrated with hot paraffin. Heart samples were embedded in paraffin blocks and 5 μm thick histology sections were prepared from the block. The sample was cut

along the long axis, and a cross section near to the center of the sample was selected, followed by Masson's trichrome staining to identify the different structures. The 2D histological images were analyzed and stitched in ImageJ (National Institutes of Health).

Correlation between histology and relaxation time maps

Due to potential tissue deformation during histological processing, the correlation analysis between histology and relaxation time maps was based on the overall size of the sinoatrial node regions identified by each modality. The observed geographical mismatch between the SAN regions in histology images and relaxation time maps likely results from the lack of spatial registration and deformation artifacts during tissue processing (Figure S2 and S3). Therefore, while the correlation between SAN sizes defined from histology and relaxation time maps is strong, it should be interpreted with this limitation in mind.

Figure S1

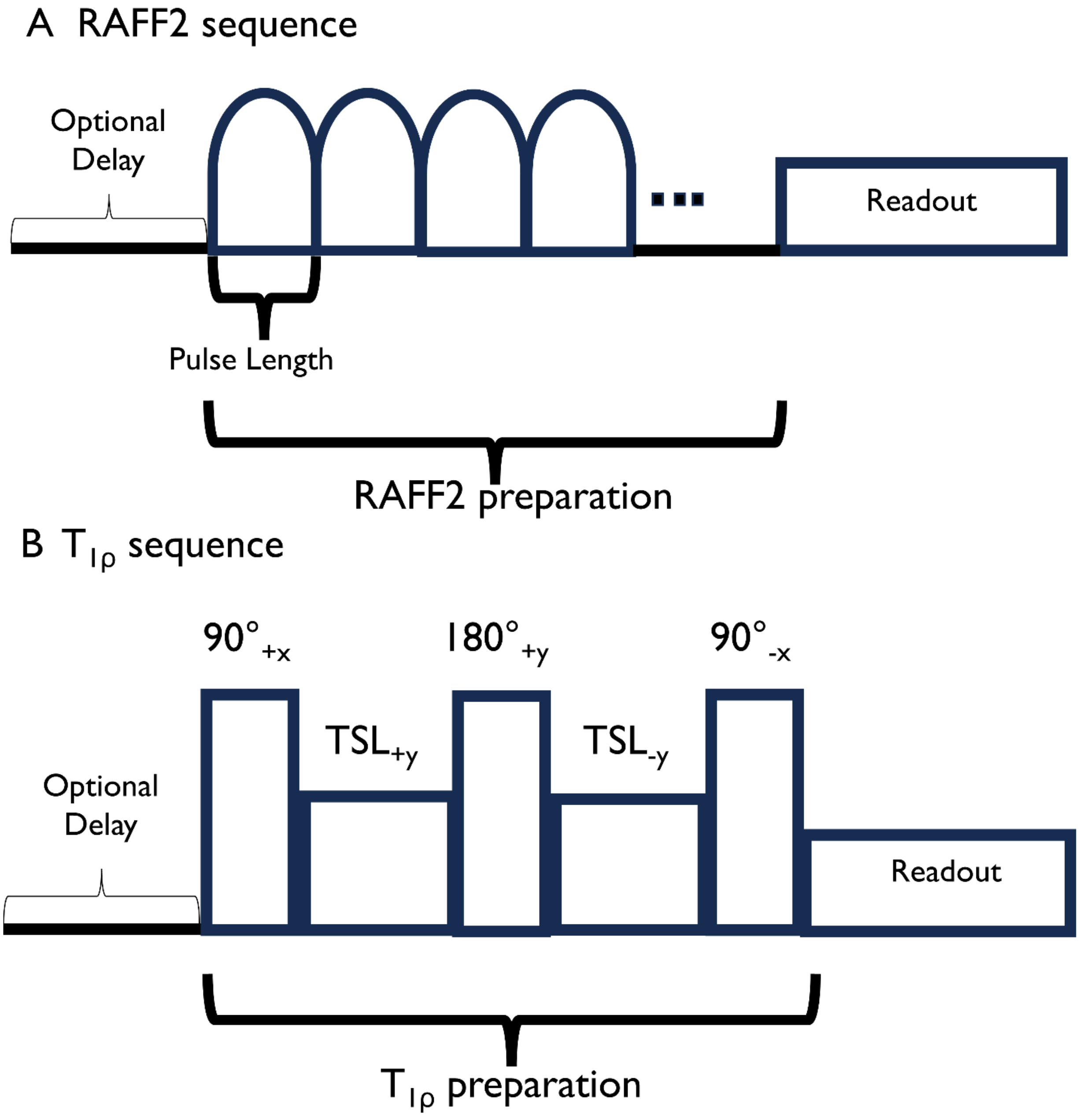

Figure S1: (A) Simplified schematic of the RAFF2 sequence, consisting of the preparation module and a read-out. (B) $T_{1\rho}$ sequence. The rotating frame preparation module consists of two 90 ° RF pulses with opposite phases and a 180 ° refocusing pulse between the spin lock halves.

Figure S2

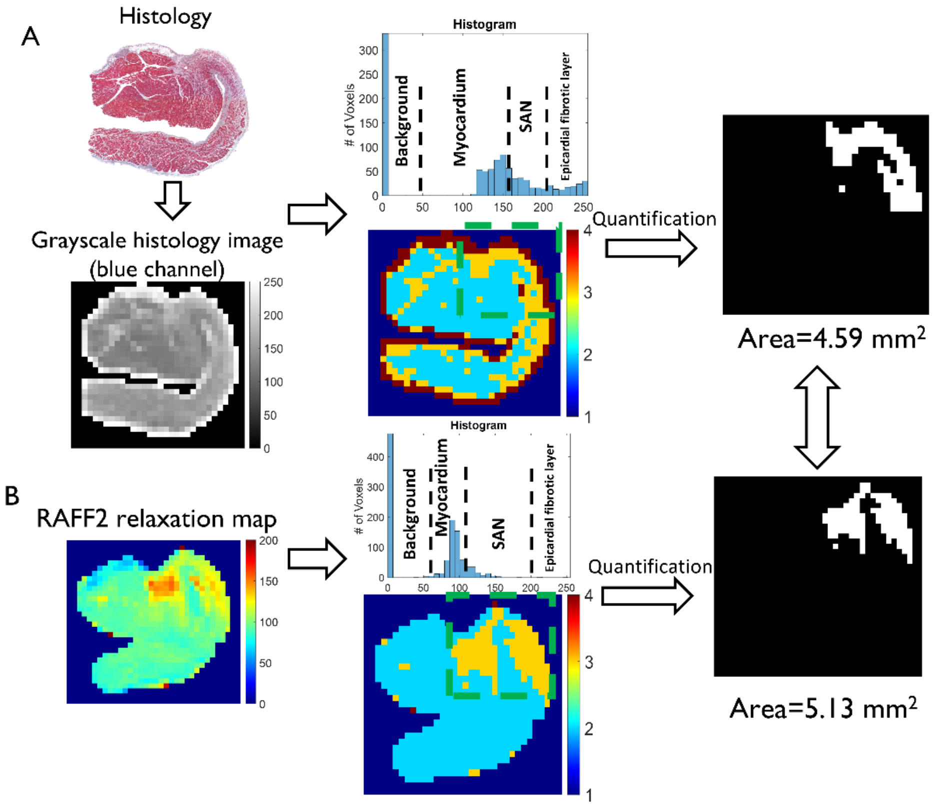

Figure S2: Correlation between the SAN areas in the histology image (A) and the corresponding RAFF2 relaxation time map (B). The blue channel of the raw RGB histology image is shown in a grayscale image. Based on the histogram of the images and optimal thresholds obtained from multi-Otsu thresholding, the pixels were divided into four classes, which are background, myocardium, SAN, and epicardial fibrotic layer. The size of SAN was quantified by the area of SAN.

Figure S3

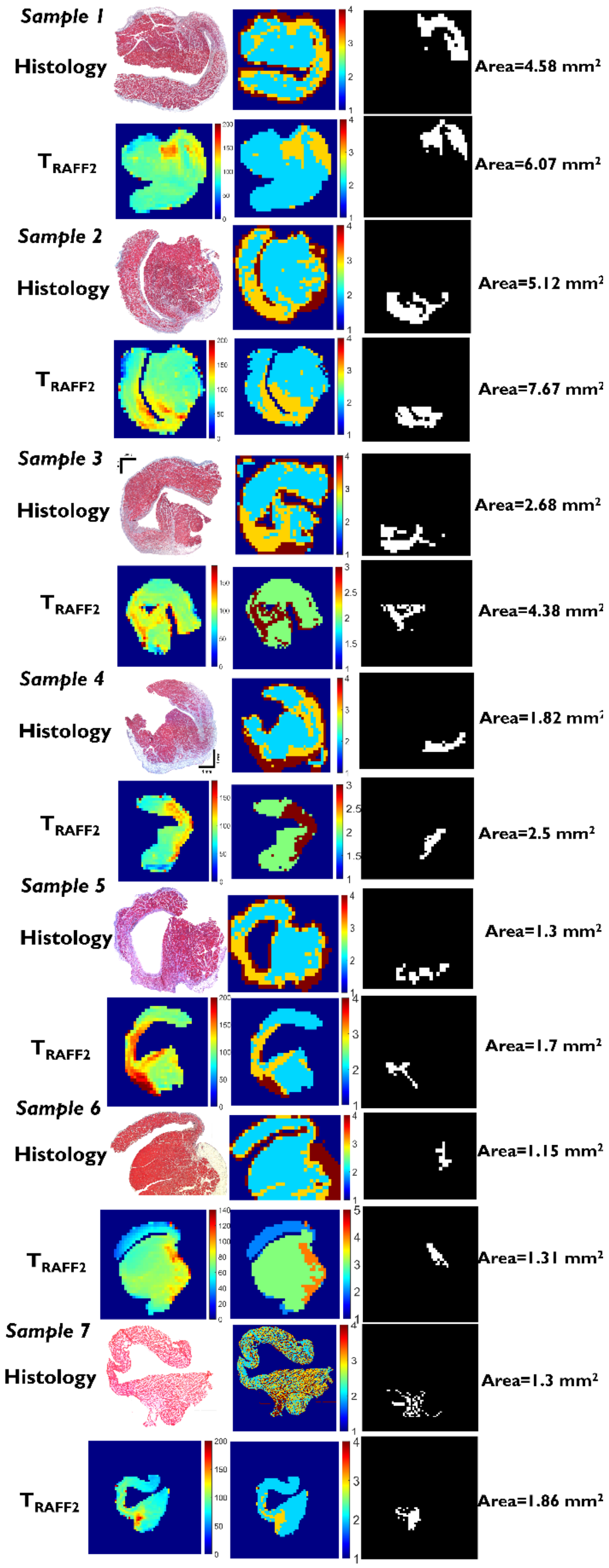

Figure S3: Histology images, corresponding T_{RAFF2} relaxation time maps, and SAN area segmentation results of seven representative samples which were scanned at 7T.

Figure S4

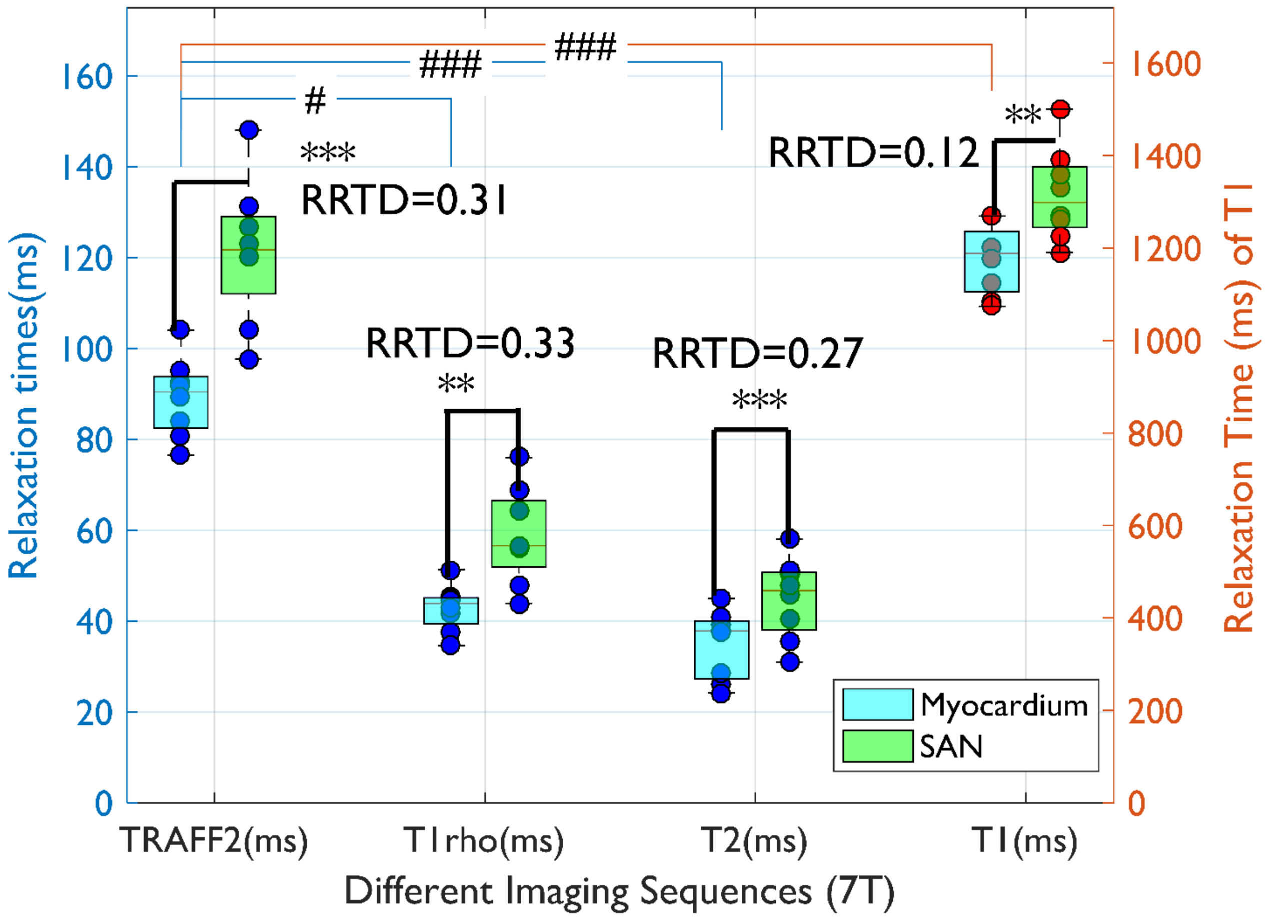

Figure S4: Relaxation times of the 7T MRI data. Contrast between SAN and myocardium was calculated by RRTD. Statistical significance (independent t-test after Benjamini-Hochberg correction) is indicated as **P<0.01, ***P<0.001 for differences in relaxation time between SAN and myocardium; and $^{\#}$P<0.05, $^{\#\#\#}$P<0.001 for differences in contrast using RAFF2 as reference.

Figure S5

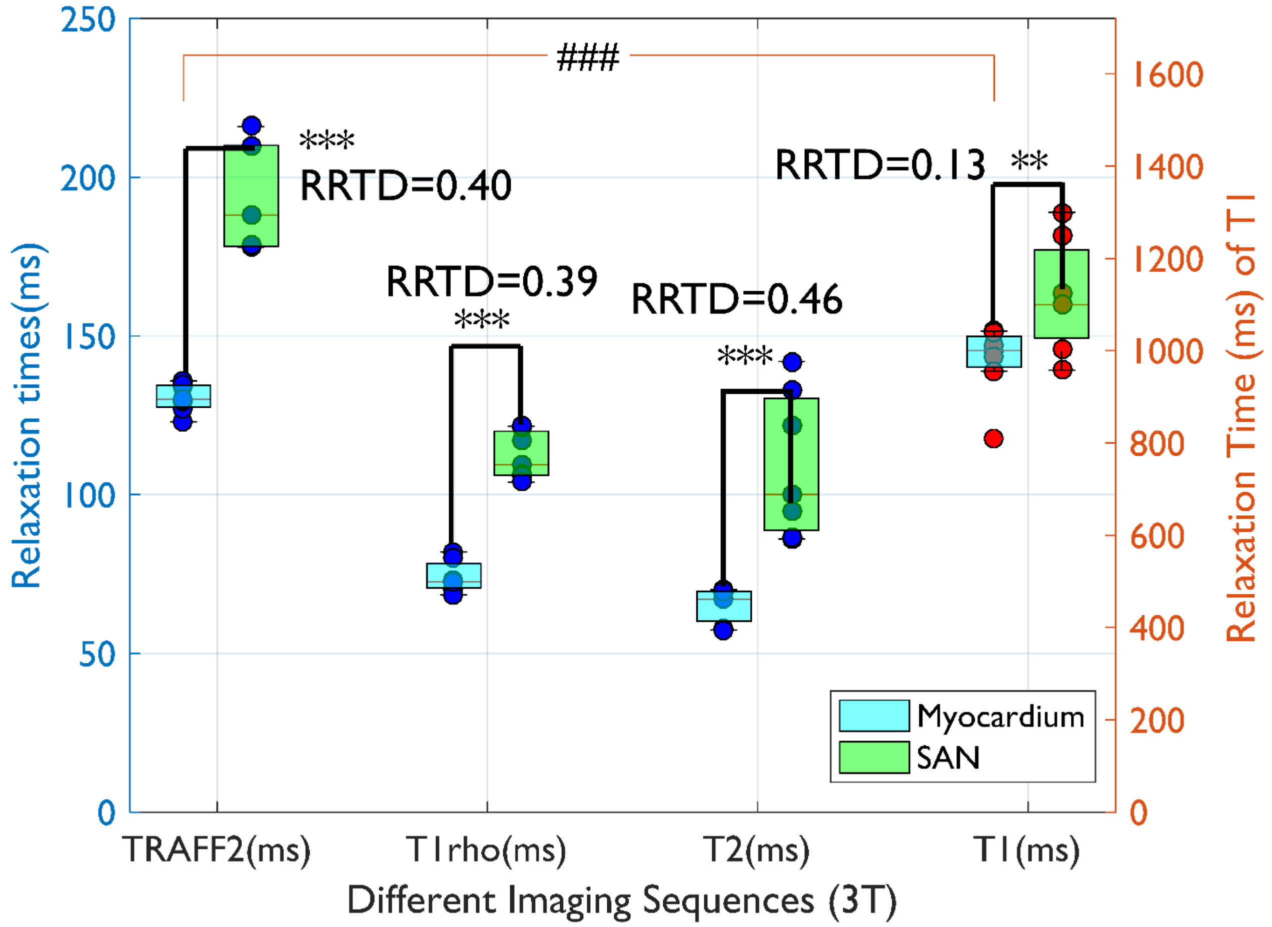

Figure S5: Relaxation times of the 3T MRI data. The contrast between SAN and myocardium was calculated using RRTD. Statistical significance (independent t-test after Benjamini-Hochberg correction) is indicated as **P<0.01, ***P<0.001 for differences in relaxation time between SAN and myocardium; and ###P<0.001 for differences in contrast using RAFF2 as a reference.

Figure S6

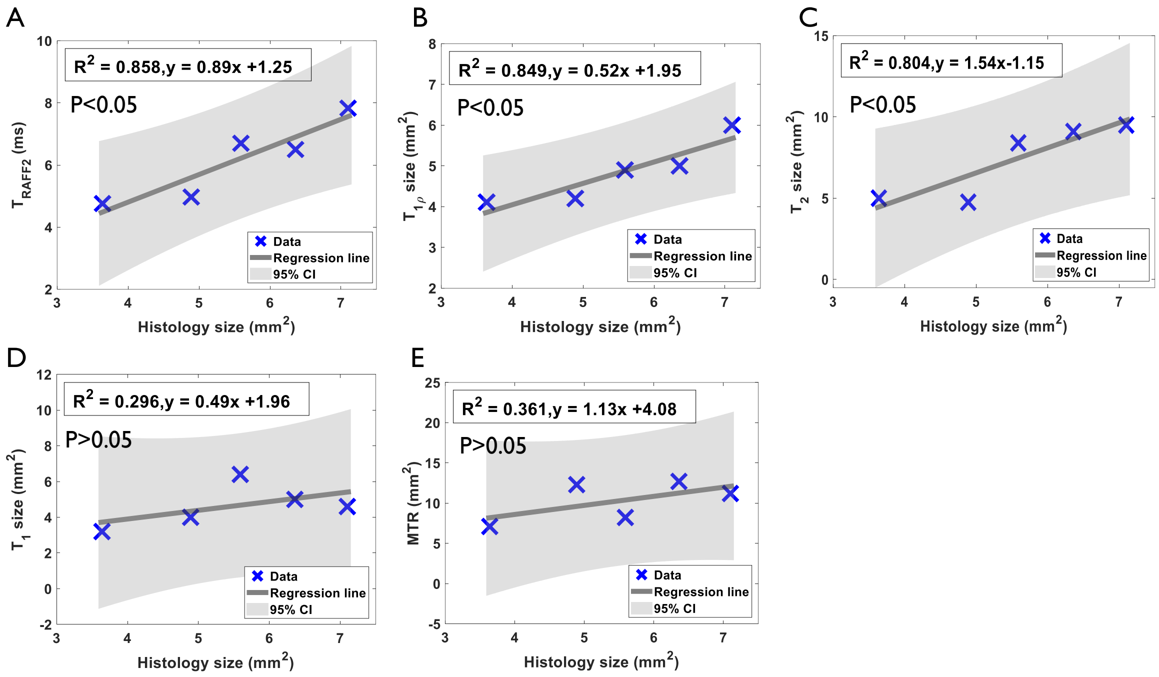

Figure S6: Linear correlation between the SAN areas determined by 3T MRI T_{RAFF2} (A), $T_{1\rho}$ (B), T_2 (C), T_1 (D), MTR (E) and Masson's trichrome stained histology sections. R^2 indicates the Pearson correlation coefficient of determination with a significance level P.

Figure S7

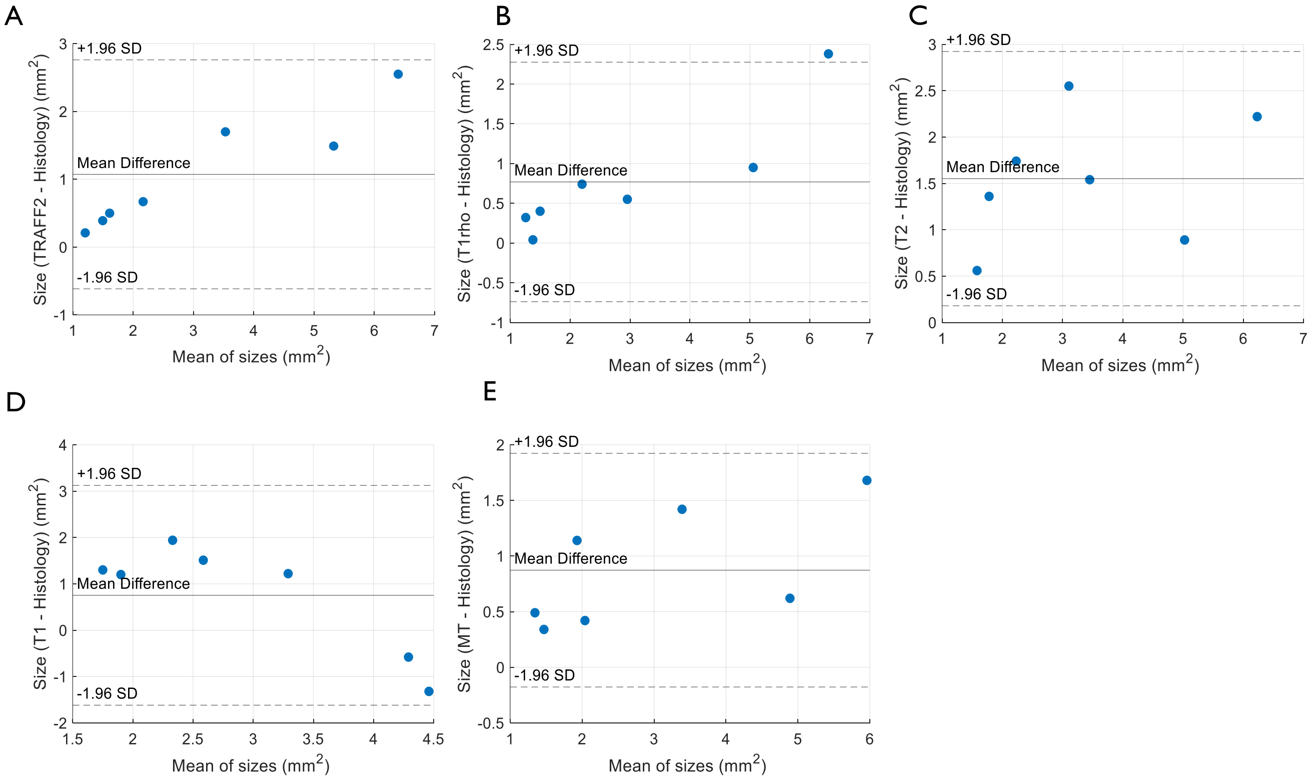

Figure S7: Bland-Altman plots showing the agreement between SAN sizes determined by histology and 7T MRI T_{RAFF2} (A), $T_{1\rho}$ (B), T_2 (C), T_1 (D), MTR (E). Each plot displays the mean difference (bias) as a solid red line and the 95% limits of agreement (mean ± 1.96 × SD) as dashed black lines.

Figure S8

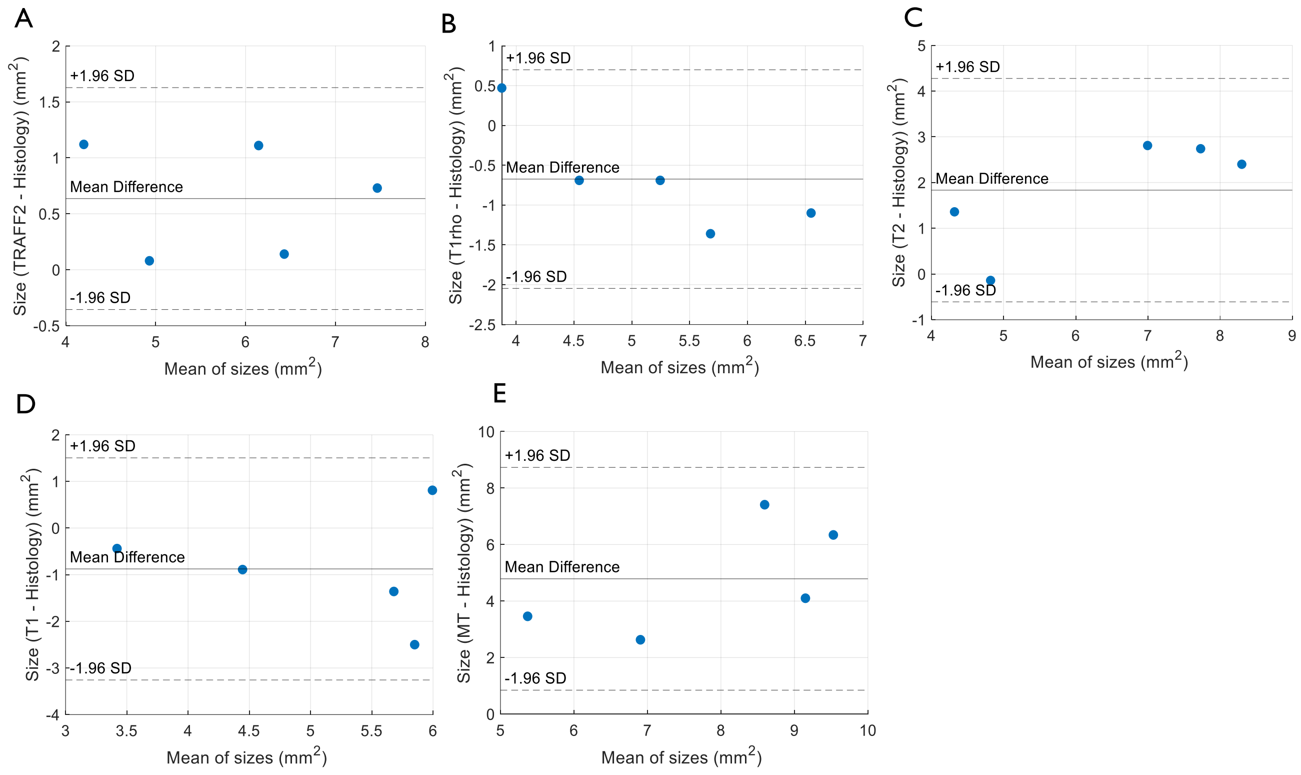

Figure S8: Bland-Altman plots showing the agreement between SAN sizes determined by histology and 3T MRI T_{RAFF2} (A), $T_{1\rho}$ (B), T_2 (C), T_1 (D), MTR (E). Each plot shows the mean difference (bias) as a solid red line and the 95% limits of agreement (mean ± 1.96 × SD) as dashed black lines.

Figure S9

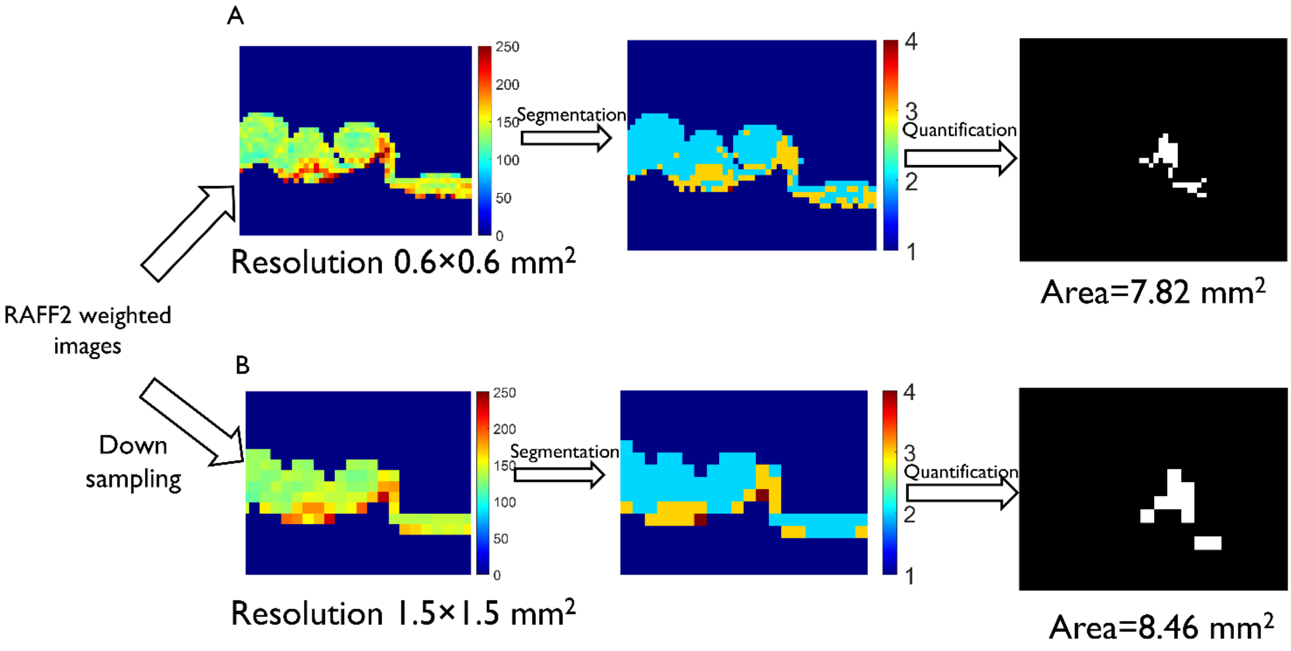

Figure S9: (A) T_{RAFF2} relaxation time map derived from high resolution (0.6 mm) RAFF2 weighted images, segmentation and quantification results of one representative sample at 3T, and (B) relaxation time map obtained from down sampling weighted images, segmentation and quantification results of the same sample.
